# Comparative Efficacy of Complement Inhibitors in Complement Inhibitor–Naïve PNH: A Systematic Review With Supportive Exploratory Network Meta‐Analysis of Randomized Trials

**DOI:** 10.1002/jha2.70250

**Published:** 2026-02-24

**Authors:** Rehan Ishaque, Abdul Subhan Talpur, Hafiza Sidra, Huda Memon, Furqan Tarique Memon, Fawad Talat, Taha Rafiq, Supratik Rayamajhi

**Affiliations:** ^1^ Liaquat University of Medical Health & Sciences Jamshoro Pakistan; ^2^ United Health Services Wilson Medical Center Johnson City New York USA; ^3^ University of Health Sciences Lahore Pakistan; ^4^ Rutgers University New Brunswick New Jersey USA; ^5^ University of Galway Galway Ireland; ^6^ Department of Internal Medicine Michigan State University East Lansing Michigan USA

**Keywords:** complement inhibitors, paroxysmal nocturnal haemoglobinuria (PNH), transfusion avoidance

## Abstract

**Introduction:**

Paroxysmal nocturnal haemoglobinuria (PNH) is an uncommon, life‐threatening disease, caused by intravascular haemolysis by the complement system. In this review, we aim to compare the efficacy of the available agents across patient‐centred outcomes in complement inhibitor‐naive patients.

**Methods:**

Following PRISMA guidelines, a comprehensive literature search was conducted on PubMed, Cochrane CENTRAL and ClinicalTrials.gov for studies published up to 30th May 2025. A frequentist model network meta‐analyses were conducted in RStudio (v5.4.1) using a common‐effects model.

**Results:**

A total of four randomized controlled trials evaluating four complement inhibitor agents (ravulizumab, crovalimab, eculizumab and pegcetacoplan) were included in this systematic review and network meta‐analysis, involving 589 complement inhibitor–naïve adults with PNH. Across outcomes, all active treatments demonstrated benefit compared with placebo or supportive care. In the exploratory network meta‐analysis of transfusion avoidance, no consistent statistically significant differences were observed between active treatments, although ravulizumab showed higher odds compared with crovalimab (OR = 2.69, 95% CI: 1.13–6.41; *p* = 0.0256). For change in FACIT‐Fatigue score, all active treatments improved fatigue versus placebo, with some statistically significant differences observed between agents; however, these comparisons were based on indirect evidence from a sparse network.

**Conclusion:**

This study suggests that available complement inhibitors improve key outcomes versus placebo/supportive care in complement inhibitors naive PNH. However, the evidence network is sparse (four trials) and the cross‐trial differences limit reliable inference regarding relative efficacy between active agents. Comparative findings should be interpreted as hypothesis‐generating.

**Abbreviations**
PNH: paroxysmal nocturnal haemoglobinuria
RCT(s): randomized controlled trial(s)
NMA: network meta‐analysis
LDH: lactate dehydrogenase
FACIT‐Fatigue: Functional Assessment of Chronic Illness Therapy—Fatigue
PRISMA: Preferred Reporting Items for Systematic Reviews and Meta‐Analyses
PROSPERO: International Prospective Register of Systematic Reviews
SAEs: serious adverse events
AEs: adverse events
GPI: glycosylphosphatidylinositol
CD55/CD59: Complement Decay‐Accelerating Factor / Membrane Attack Complex Inhibitor (GPI‐anchored proteins)
ULN: upper limit of normal
MD: mean difference
CI: confidence interval
OR: odds ratio
SD: standard deviation
NA: not available
ROB: risk of bias
SUCRA: surface under the cumulative ranking curve
RBC: red blood cells

## 1 Introduction

Paroxysmal nocturnal haemoglobinuria (PNH) is a rare and deadly haematologic disorder caused by a lack of the glycosylphosphatidylinositol GPI‐anchored proteins CD55 and CD59 on the surface of haematopoietic cells [1]. Lack of these proteins makes red blood cells more vulnerable to the terminal complement system's attack, which can lead to intravascular haemolysis, anaemia, exhaustion, thrombosis, renal insufficiency and eventually even death [2]. If treatment is not received, the condition can lead to severe morbidity and mortality, with thrombotic complications being the leading cause of death for those who are impacted [3]. Fortunately, the development of complement inhibitors (such as eculizumab, ravulizumab, pegcetacoplan and crovalimab) has substantially enhanced the prognosis of patients with PNH. The first to receive a license for use in clinical settings was eculizumab [4].The risk of thrombotic episodes and the need for transfusions were considerably decreased by a monoclonal antibody that mainly targets complement factor 5 (C5) [5]. Since then, additional complement inhibitors have been developed, each with unique pharmacokinetic characteristics, modes of action and administration methods. Pegcetacoplan is a C3 inhibitor with proximal complement blockade [6], ravulizumab is a C5 inhibitor with an extended half‐life and crovalimab is a C5 inhibitor that is administered subcutaneously [7].

Despite therapeutic advances, selecting among available complement inhibitors remains challenging. No randomized head‐to‐head trials comparing all approved agents in complement inhibitor‐naive PNH patients have been conducted to date. Instead, individual agents have been evaluated in separate randomized trials against placebo or an active comparator, limiting direct comparative inference [5]. Moreover, treatment decisions in PNH extend beyond haemolysis control and include patient‐centred outcomes such as transfusion avoidance, haemoglobin stabilization, quality of life and treatment convenience [7]. Differences in trial design, baseline patient characteristics and outcome definitions further complicate cross‐trial comparisons and interpretation of relative efficacy.

In this study, we aim to combine data from several randomized controlled trials (RCTs) using a common comparator and to conduct a systematic review with a supportive exploratory network meta‐analysis (NMA), enabling indirect comparisons across all available agents, in contrast to conventional pairwise meta‐analyses that are constrained by the absence of direct comparisons [1]. This analysis aims to summarize current data on the relative effects of approved complement inhibitors across clinically relevant outcomes. Given the limited number of trials and the absence of direct comparisons, the findings are intended to be exploratory and hypothesis‐generating, highlighting areas where further comparative research is needed.

## 2 Methods

### 2.1 Data Sources and Search Strategy

The literature search for this systematic review and meta‐analysis was performed according to the guidelines outlined in Preferred Reporting Items for Systematic Reviews and Network Meta‐Analysis (PRISMA) [8]. Three electronic databases (PubMed, Cochrane Library and ClinicalTrials.gov) were searched from the date of inception to 30th May, 2025, using the MeSH terms and keywords for ‘paroxysmal nocturnal haemoglobinuria’ AND ‘complement inhibitors,' ‘eculizumab,’ ‘ravulizumab,’ ‘pegcetacoplan’ AND ‘outcomes’ to identify eligible studies focusing complement inhibitor naive patients. Therefore, validated RCT filters were applied to narrow down the search to RCTs. The search strategy was designed to support a systematic review of randomized evidence, with NMA planned as an exploratory secondary synthesis. Given the objective to synthesize high‐quality comparative evidence, we restricted inclusion to only double‐arm RCTs. No publication time limits were applied. Our literature research produced a total of 380 results. All 380 search results were imported into the Rayyan web version for systematic screening [9].

### 2.2 Selection Criteria

**FIGURE 1:**
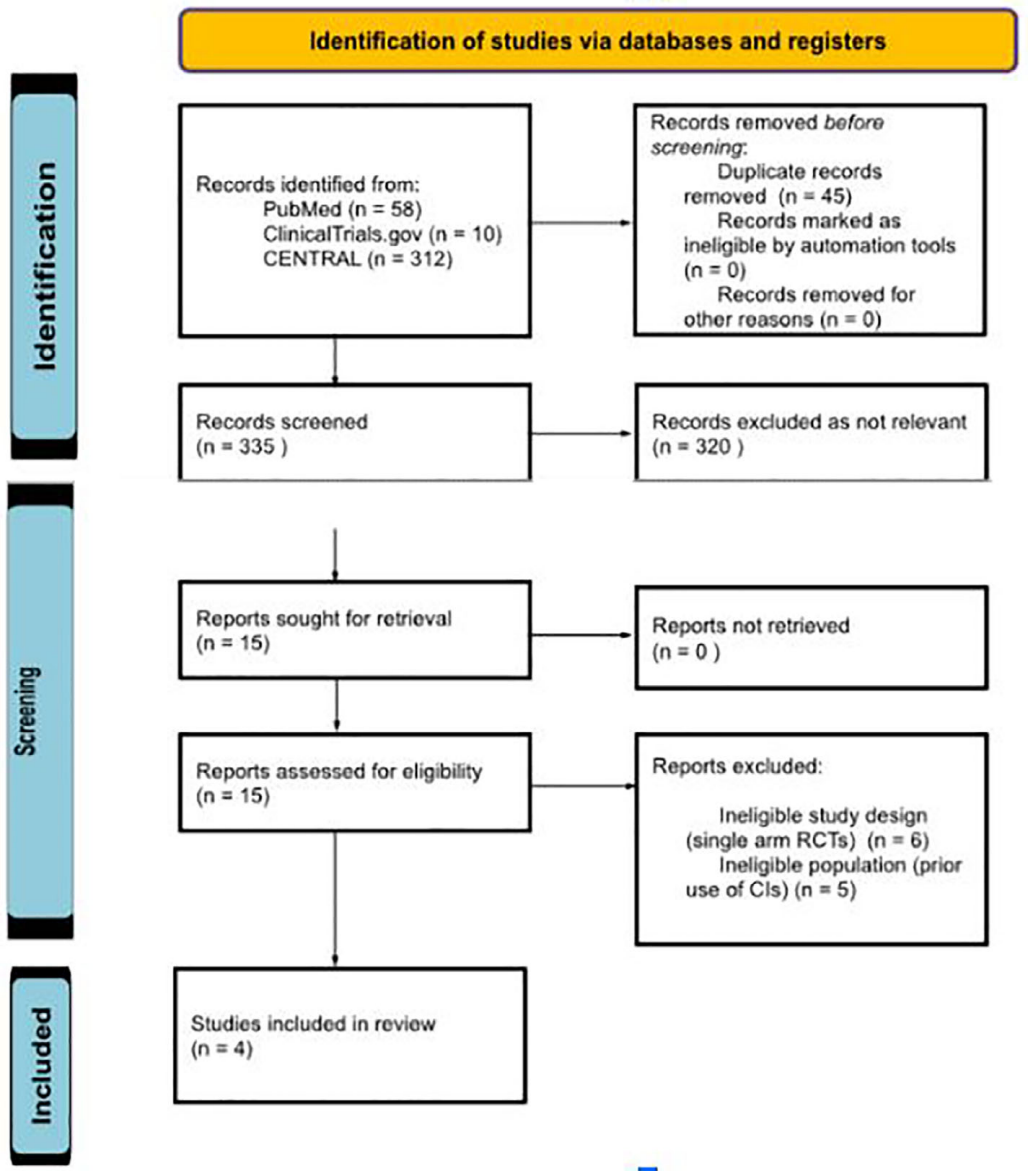
PRISMA flow diagram for the selection of studies.

After removing 45 duplicates, the remaining 335 articles were screened independently by two authors. In primary screening, 320 non‐relevant articles were excluded based on their titles and abstracts. Full texts of the remaining 15 articles were assessed for eligibility based on predetermined criteria. Of these, four articles met the inclusion criteria and were included in the systematic review and exploratory NMA. A PRISMA 2020 flow diagram is presented in Figure 1. The remaining studies were excluded because they did not exclusively involve complement inhibitor naive patients or lacked a comparator arm (i.e., single‐arm studies or crossover designs). Inclusion criteria were: (1) double arm RCT, (2) studies using complement inhibitors as an intervention and (3) studies with comparator arm including either placebo or an active complement inhibitor comparator drug. Exclusion criteria included (1) review articles, case reports, case series, observational studies, conference abstracts and commentaries, (2) studies with no comparator arm, (3) studies with predominant paediatric patient population and (4) studies with participants that had previously used complement inhibitors for treating PNH and are not complement inhibitor naive. The inclusion and exclusion criteria is further detailed in Table 1.

**TABLE 1:** Inclusion and exclusion criteria for the primary and secondary screening process.

Criteria	Inclusion	Exclusion
**Study Design**	Only double‐arm randomized controlled trials (RCTs)	Non‐randomized trials, observational studies, case reports, reviews, conference abstracts
**Population**	Paroxysmal nocturnal haemoglobinuria (PNH), complement inhibitor–naïve patients	Patients with prior treatment experience with complement inhibitors
**Interventions**	Pegcetacoplan, crovalimab, ravulizumab, eculizumab	Treatments other than the complement inhibitors.
**Comparators**	Other complement inhibitor agents (e.g., eculizumab, ravulizumab) or placebo/supportive care	Single‐arm RCTs
**Outcomes (efficacy)**	Any of the following: Change in LDH levels Transfusion avoidance Haemoglobin stabilization Breakthrough haemolysis FACIT‐fatigue score	Studies not reporting any of the given outcomes.
**Outcomes (safety)**	Adverse events (AEs), Serious adverse events (SAEs)	Safety outcomes not reported.
**Language**	Studies published in English	Non‐English publications
**Population characteristics**	Adult patients (≥ 18 years)	Paediatric only studies.

### 2.3 Data Extraction

Two authors independently extracted data from the four included studies. Following individual data extraction, data sheets were double‐checked by another author for any discrepancies. The data were collected for baseline characteristics, including sex, race (Asian, Black/African American, White), weight (median, range), central LDH (mean, SD, ×ULN), haemoglobin (median, range) and number of patients who received ≥ 1 unit of packed RBCs in the 12 months prior to screening; comorbidities including aplastic anaemia, major vascular events and myelodysplastic syndrome; as well as laboratory parameters such as erythrocytes, granulocytes and monocytes. Efficacy and safety outcomes assessed at the primary endpoint timepoint (26 weeks) included haemolysis control (OR), transfusion avoidance (absolute difference), breakthrough haemolysis (percentage difference), haemoglobin stabilization (percentage difference), FACIT‐fatigue score (mean difference in points) and LDH normalization. We adhered to the definitions and categories used in individual studies without applying external classifications.

### 2.4 Risk of Bias Assessment

The Cochrane RoB tool 2.0 was employed for risk of bias assessment of RCTs. This tool assesses the methodological quality of randomized trials across five key domains, including the randomization process, deviations from intended interventions, missing outcome data, outcome measurement and selection of reported results. Studies were then rated as low risk, some concerns or high risk of bias [10] (Supporting Information S1).

### 2.5 Data Analysis

In order to compare the effectiveness and safety of available complement‐targeted therapies in complement inhibitor‐naive patients with PNH, we performed a frequentist NMA using the netmeta package in Rstudio (version 4.5.1) [11]. Given the limited number of included trials and similar follow‐up duration, a common‐effects model was used, acknowledging that residual clinical heterogeneity could not be formally explored.

Four RCTs assessing pegcetacoplan, crovalimab, ravulizumab and eculizumab were part of the network as the intervention group. Other active complement inhibitors or a placebo were used as comparators. In order to enable indirect comparisons between treatment nodes, the network was built using shared comparators, specifically eculizumab and placebo [12]. To ensure baseline comparability across study populations, adult PNH patients who had never taken a complement inhibitor were enrolled in each RCT. Furthermore, all outcomes were assessed at a common timepoint of 26 weeks to facilitate consistent data synthesis across studies [6, 13, 14, 15]. We looked at efficacy endpoints which involved breakthrough haemolysis, lactate dehydrogenase (LDH) normalization, haemoglobin stabilization, transfusion avoidance and an improvement in the FACIT‐fatigue score [16].

Effect estimates were presented using comparative forest plots and league tables [11]. It was not possible to conduct a formal statistical test for inconsistency (such as the design‐by‐treatment interaction model) because of the limited number of included studies (*n* = 4) and the lack of closed loops in the network geometry [17]. However, because patient populations, baseline characteristics, study designs and outcome definitions were similar across trials to a certain extent, the assumption of transitivity was considered plausible, although it could not be formally tested due to the sparse network [18]. All indirect comparisons between active treatments should be interpreted as exploratory, as the network was sparse and lacked closed loops required for formal inconsistency assessment.

## 3 Results

### 3.1 Baseline Demographics

A total of 4 RCTs evaluating four complement inhibitor agents (ravulizumab, crovalimab, eculizumab and pegcetacoplan) were included in this meta‐analysis, involving 589 complement inhibitor–naïve adults with PNH. The age of the participants ranged from 17 to 78. The proportion of male patients was 52.6% (*n* = 310/589). The weight of the participants ranged from 42 to 142.3 kg. Among 502 patients across three trials, 60.8% were Asian (*n* = 305/502), 2.3% were Black (*n* = 12/502) and 18.7% were White (*n* = 94/502). A total of 353 out of 502 patients (70.3%) received ≥ 1 unit of packed red blood cells in the 12 months prior to screening. Across the included studies, 97 of 290 patients (33.44%) had a history of aplastic anaemia, 48 of 290 (16.5%) had a history of major vascular events (3 studies) and 14 of 290 (4.8%) had myelodysplastic syndrome (2 studies). The baseline haemoglobin levels of the participants ranged from 58 to 135 g/L. PNH clone size at baseline ranged from 0.1%–96.0% in erythrocytes, 5.8%–100% in granulocytes and 41.5%–100% in monocytes across the included studies. Every trial reported outcomes at a uniform 26‐week timepoint. Baseline characteristics varied across trials, including differences in the prevalence of bone marrow failure syndromes and prior transfusion exposure, particularly in COMMODORE 2. Among the studies for which ROB assessment was available, there was: (1) low ROB in TRIUMPH trial (2) moderate ROB in PRINCE, COMMDORE 2 and ALXN1210. Table 2 provides a full description of the baseline characteristics of the included studies.

**TABLE 2:** Baseline characteristics of the study participants.

	COOMDORE 2		TRIUMPH		PRINCE		ALXN1210	
Variable	Crovalimab	Eculizumab	Eculizumab	Supportive	Pegcetacoplan	Supportive	Ravulizumab	Eculizumab
**Sample size**	134	69	43	44	35	18	125	121
**Age (median range)**	36 (18–76)	38 (17–78)	41 (20–85)	35 (18–78)	42.2 (22–67)	49.1 (20–74)	44.8 ± 15.2	46.2 ± 16.2
**Male *n* (%)**	77 (57.0)	35 (50.7)	20	15	19 (54.3%)	10 (55.6%)	65 (52.0)	69 (57.0)
**Asian *n* (%)**	86 (63.7)	51 (73.9)	NA	NA	23 (65.7%)	16 (88.9%)	72 (57.6)	57 (47.1)
**Black/African American *n* (%)**	3 (2.2)	1 (1.4%)	NA	NA	2 (5.7%)	0	2 (1.6%)	4 (3.3%)
**White *n* (%)**	45 (33.3%)	16 (23.2%)	NA	NA	0	0	43 (34.4)	51 (42.1)
**Weight, median (range), kg**	66 (42.0‐140.3)	62 (47.0‐122.0)	NA	NA	NA	NA	68.2 ±15.6	69.2 ± 14.9
**Haemoglobin, median (range), g/L‡**	85.0 (63.0‐135.0)	87.0 (58.0‐810.0)	NA	NA	9.4 ± 1.4	8.7 ± 0.8	NA	NA
**Packed RBC units transfused in the past 12 months prior to screening,** **≥ 1 unit, *n* (%)**	103 (77.4)	50 (73.5)	NA	NA	29 (82.9)	14 (77.8%)	79 (63.2%)	78 (64.5%)
**History of aplastic anaemia *n* (%)**	53 (39.3)	26 (37.7)	6 (14%)	12 (27%)	5 (14.3%)	5 (27.8%)	NA	NA
**Major vascular events *n* (%)**	21 (15.6)	10 (14.5)	9 (21%)	8 (18%)	NA	NA	17 (13.6%)	25 (20.7%)
**Myelodysplastic syndrome *n* (%)**	6 (4.4)	6 (8.7)	2 (5%)	0 (0%)	NA	NA	NA	NA

**FIGURE 2:**
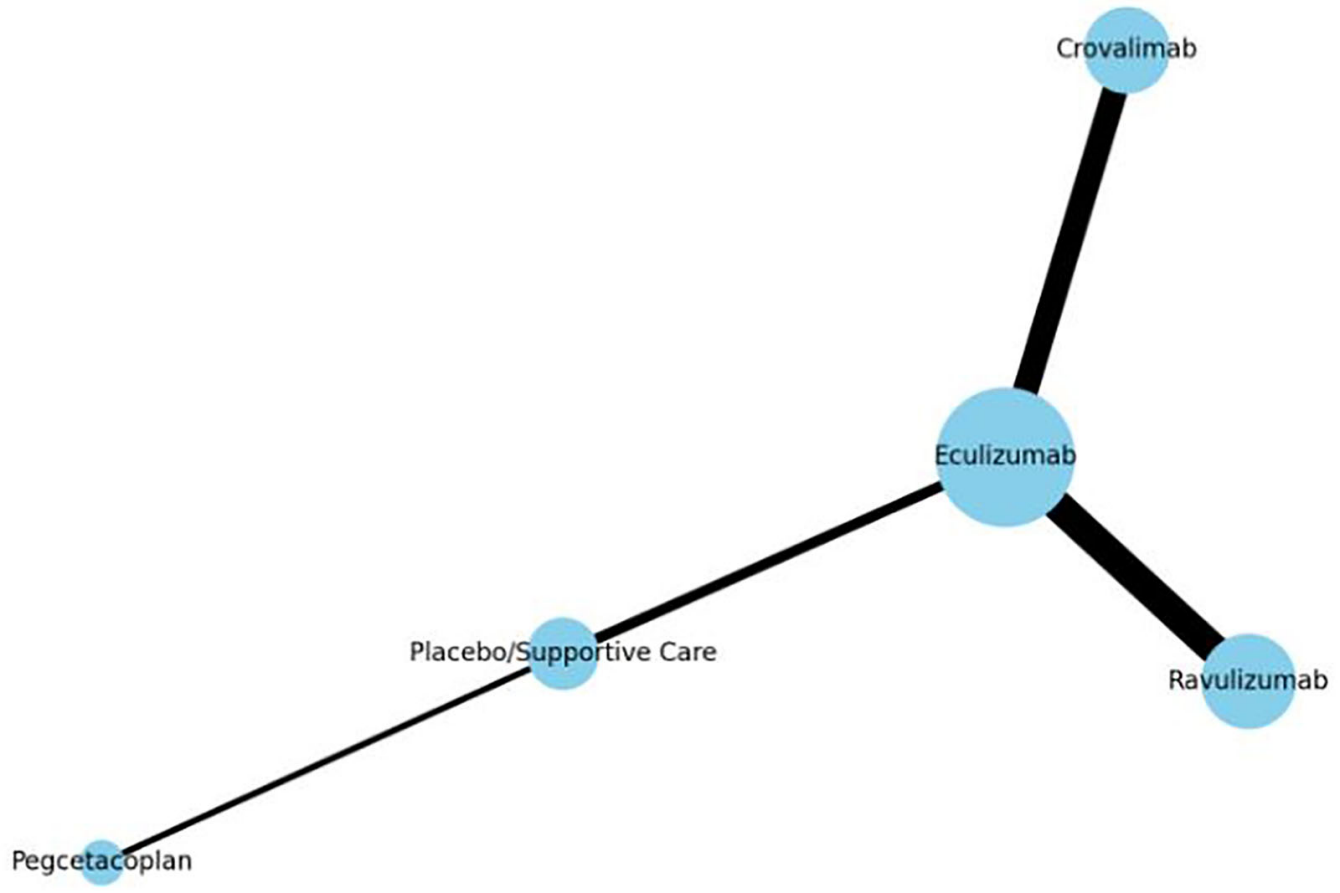
Network plot (nodes represent treatment arms [e.g., eculizumab, ravulizumab], node size is proportional to the number of patients involved, edge thickness is proportional to the number of patients in each direct comparison, layout is spaced to ensure clarity and minimize overlap).

Figure 2 shows a network plot for all included treatment comparisons. Every treatment is represented by a node, and the size of each node varies according to the number of patients assigned to that treatment at random. Each connecting line and its thickness have significance as well. Each connecting line symbolizes a direct comparison made in a RCT, and since only one trial informed each comparison, the thickness of the lines also reflects the sample size of the corresponding trial instead of the number of studies.

### 3.2 Transfusion Avoidance

**FIGURE 3:**
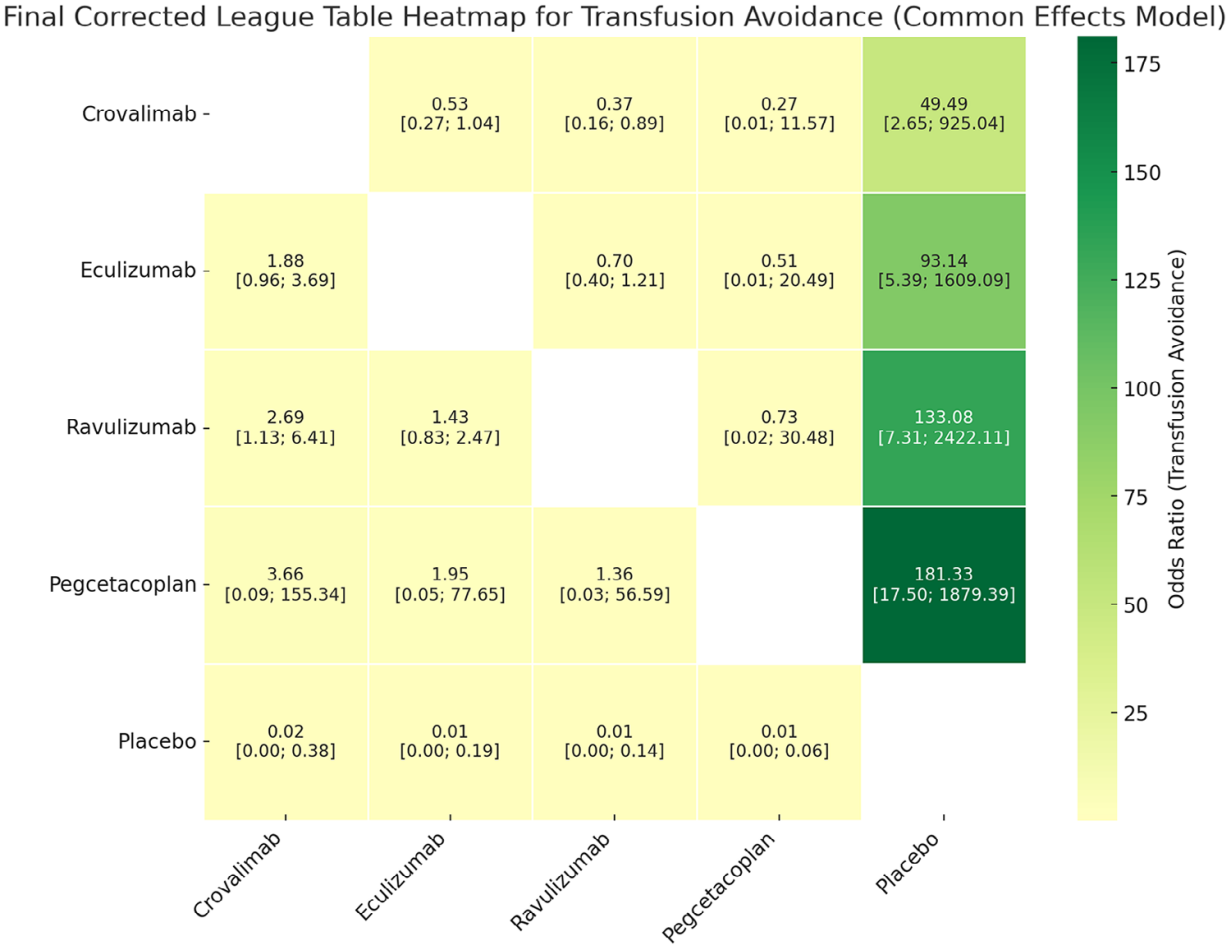
Table for transfusion avoidance.

In this exploratory NMA of four RCTs, all active treatments were associated with higher odds of transfusion avoidance compared with placebo. Pegcetacoplan demonstrated the largest point estimate as compared to placebo (OR = 181.33, 95% CI: 17.50–1879.39), followed by ravulizumab (OR = 133.08, 95% CI: 7.31–2422.11), eculizumab (OR = 93.14, 95% CI: 5.39–1609.09) and crovalimab (OR = 49.49, 95% CI: 2.65–925.04). Pairwise comparisons between active treatments showed overlapping confidence intervals in most cases; However, ravulizumab showed a statistically significant advantage over crovalimab (OR = 2.69, 95% CI: 1.13–6.41; *p* = 0.0256) (Figure 3). It is also important to note that transfusion avoidance is strongly influenced by baseline transfusion burden, which differed substantially across trials and could not be adjusted for. As a result, indirect comparisons for this outcome should be interpreted with caution and considered exploratory rather than definitive.

### 3.3 Haemoglobin Stabilization

**FIGURE 4:**
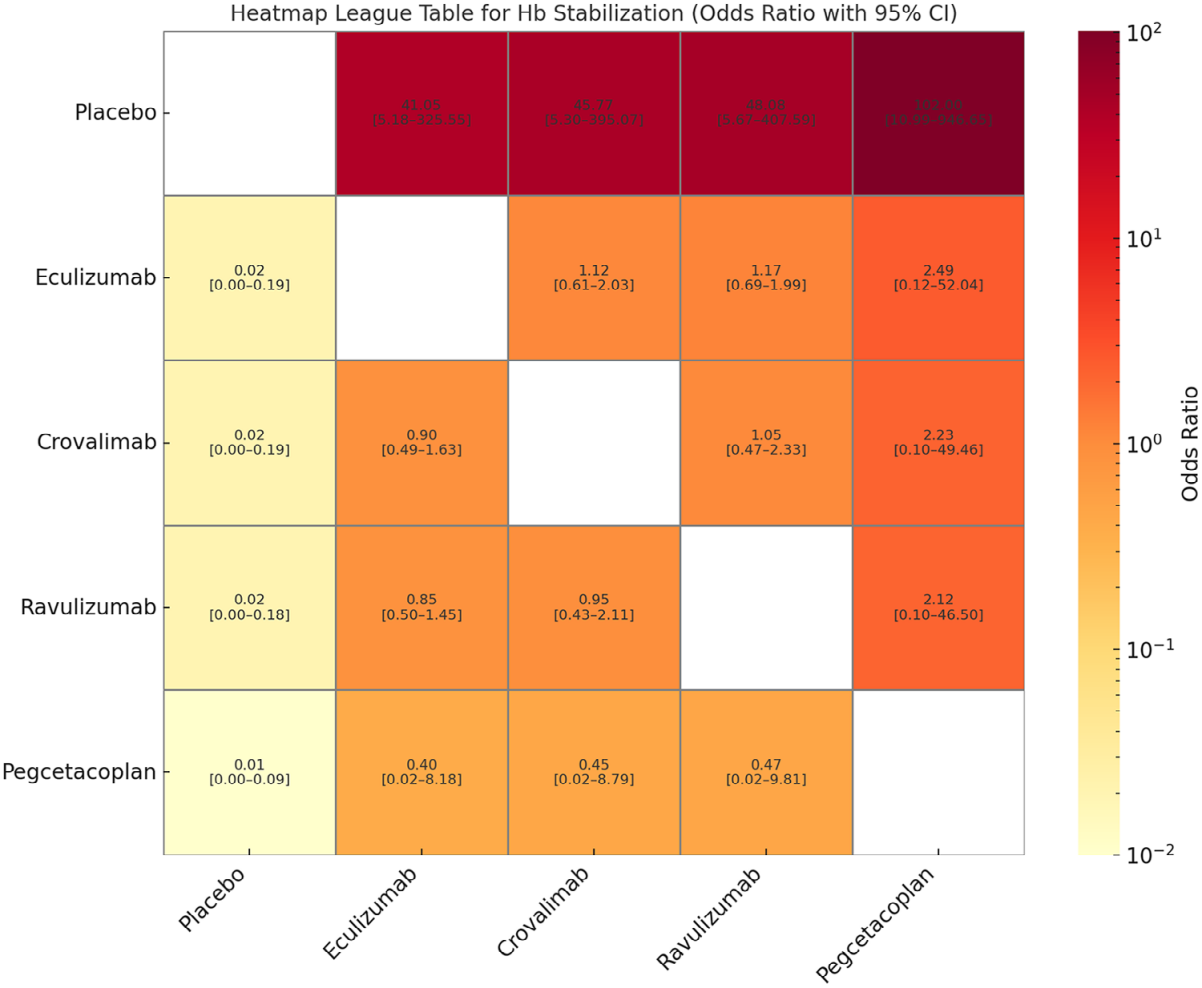
Table for Hb stabilization.

In the NMA of four RCTs evaluating haemoglobin stabilization, all active treatments (crovalimab, eculizumab, ravulizumab and pegcetacoplan) demonstrated statistically significant superiority over placebo. The highest odds were observed with pegcetacoplan (OR = 102.00, 95% CI: 10.99–946.65), followed by ravulizumab (OR = 48.08, 95% CI: 5.67–407.59), crovalimab (OR = 45.77, 95% CI: 5.30–395.07) and eculizumab (OR = 41.05, 95% CI: 5.18–325.55). Despite these large point estimates against placebo, no statistically significant differences were observed between the active treatments themselves. The wider confidence intervals are likely due to the very small number of participants with haemoglobin stabilization in the placebo group, very close to zero (Figure 4).

### 3.4 LDH Normalization

**FIGURE 5:**
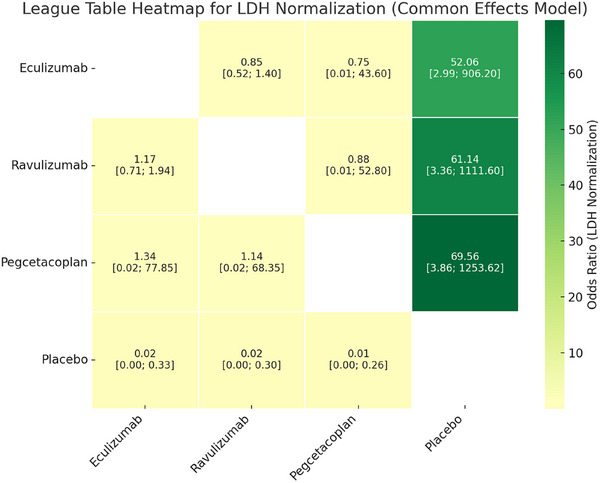
Table for LDH normalization.

In the NMA of four RCTs evaluating LDH normalization, all active treatments reported statistically significant benefit over placebo. The pooled effect estimates of pegcetacoplan compared to placebo were (OR = 69.56, 95% CI: 3.86–1253.62), followed closely by ravulizumab (OR = 61.14, 95% CI: 3.36–1111.60) and eculizumab (OR = 52.06, 95% CI: 2.99–906.20). The wider CI is likely due to the very small number of events in the placebo group. However, no statistically significant differences were observed between active treatments in indirect comparisons (Figure 5).

### 3.5 FACIT‐Fatigue Score

In the NMA of four RCTs evaluating change in FACIT‐Fatigue scores, all active treatments demonstrated improvement over placebo. Crovalimab showed the largest mean improvement (MD = +13.00, 95% CI: 8.46–17.54), followed by ravulizumab (MD = +11.07, 95% CI: 6.56–15.58), eculizumab (MD = +10.40, 95% CI: 6.30–14.50) and pegcetacoplan (MD = +4.50, 95% CI: –0.20 to 9.20). Improvements with crovalimab, ravulizumab and eculizumab exceeded the established minimum clinically important difference for FACIT‐Fatigue (approximately three to five points), suggesting clinically meaningful benefit versus placebo.

**FIGURE 6:**
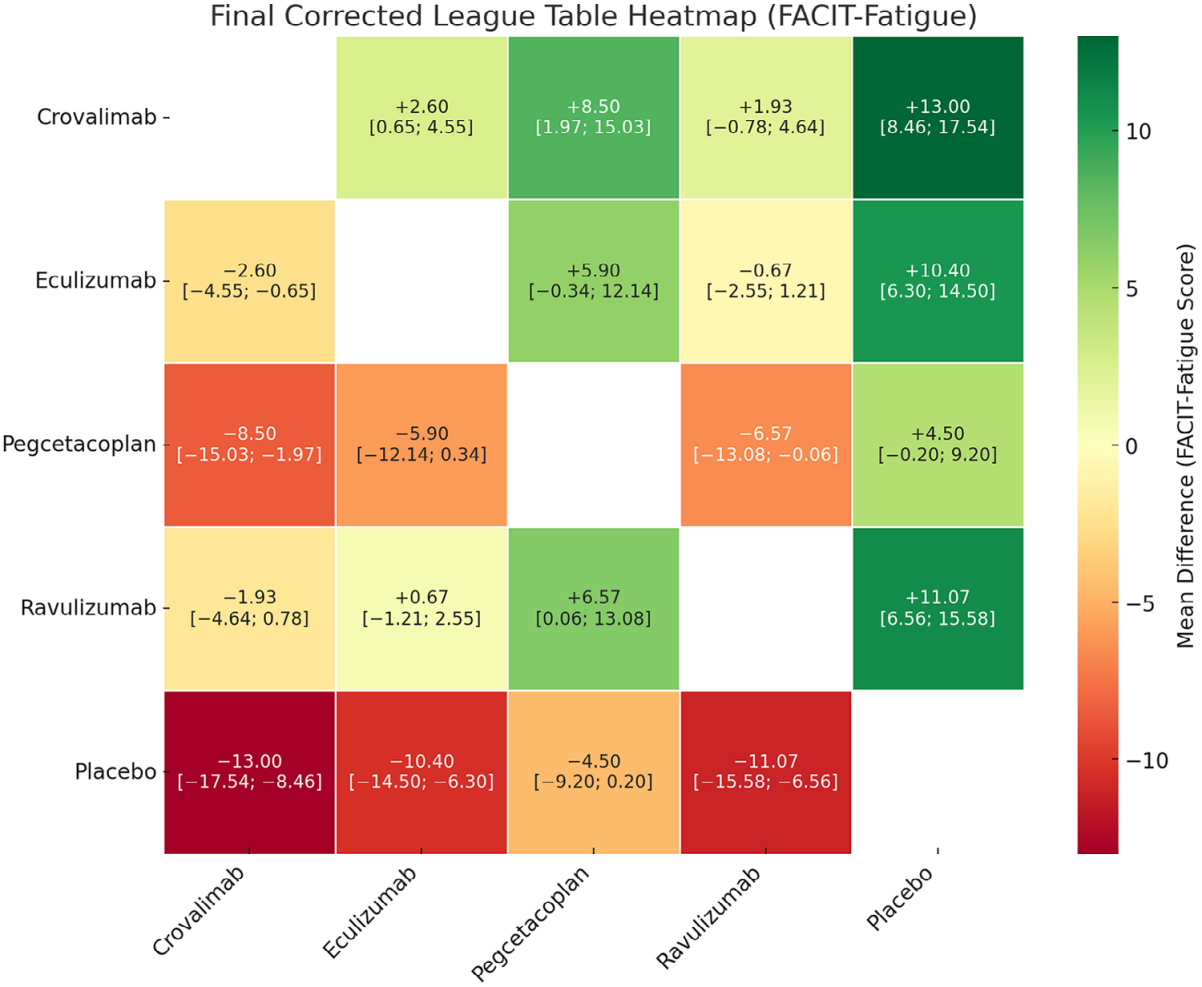
Table for FACIT fatigue score.

Upon comparison of active treatments with each other, crovalimab demonstrated significantly greater improvement compared to pegcetacoplan (MD = +8.50, 95% CI: 1.97–15.03) and eculizumab (MD = +2.60, 95% CI: 0.65–4.55). However, there was no statistically significant difference found when compared to ravulizumab (MD = +1.93, 95% CI: –0.78 to 4.64). Ravulizumab also showed greater improvement than pegcetacoplan (MD = +6.57, 95% CI: 0.06–13.08). Given the exploratory nature of the network and reliance on indirect evidence, these differences should be interpreted cautiously (Figure 6).

Funnel plots, publication bias, sensitivity analysis and subgroup analysis were not feasible due to the limited number of studies.

#### 3.5.1 Secondary Outcomes

**FIGURE 7:**
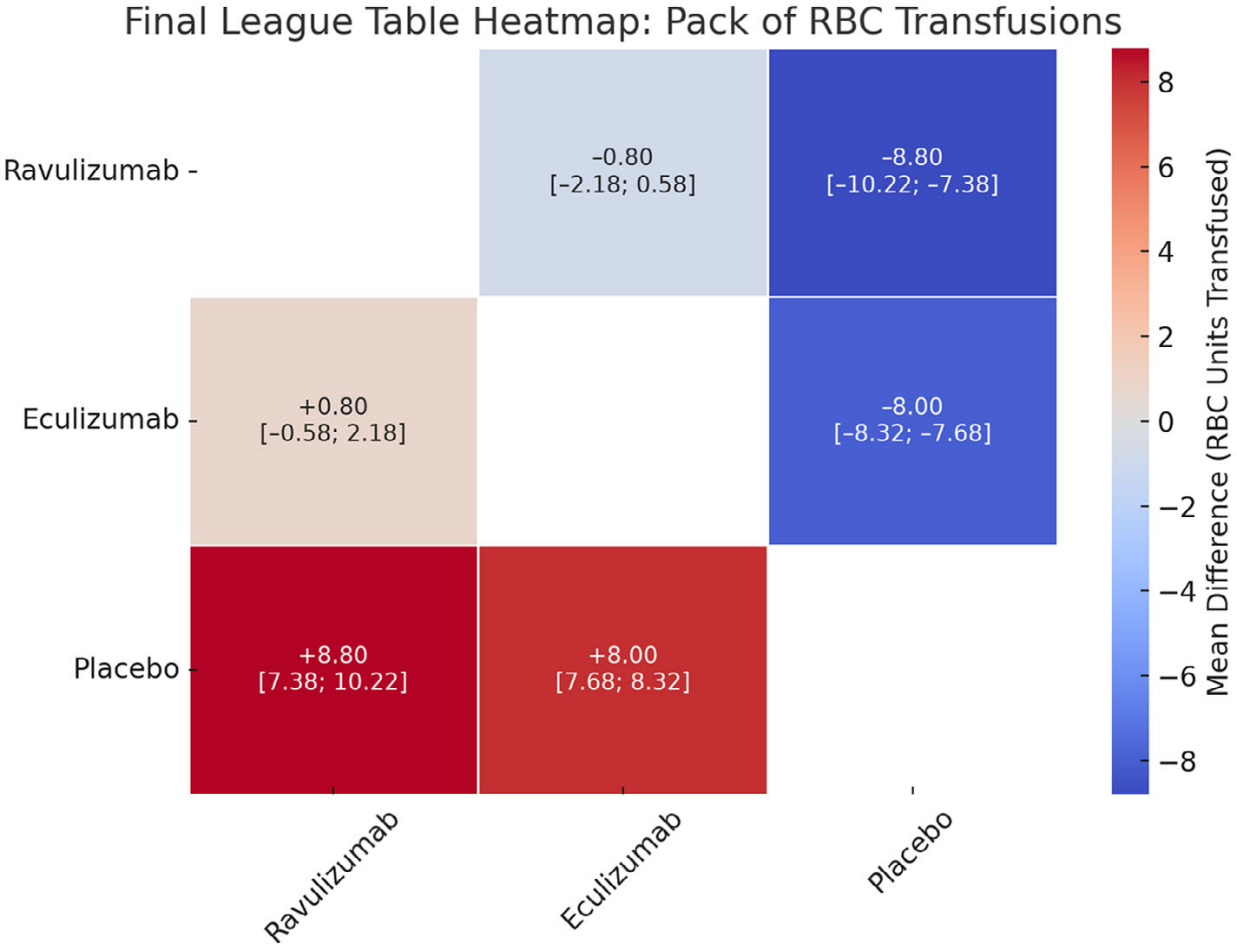
Table for packs of RBCs transfusions.

**FIGURE 8:**
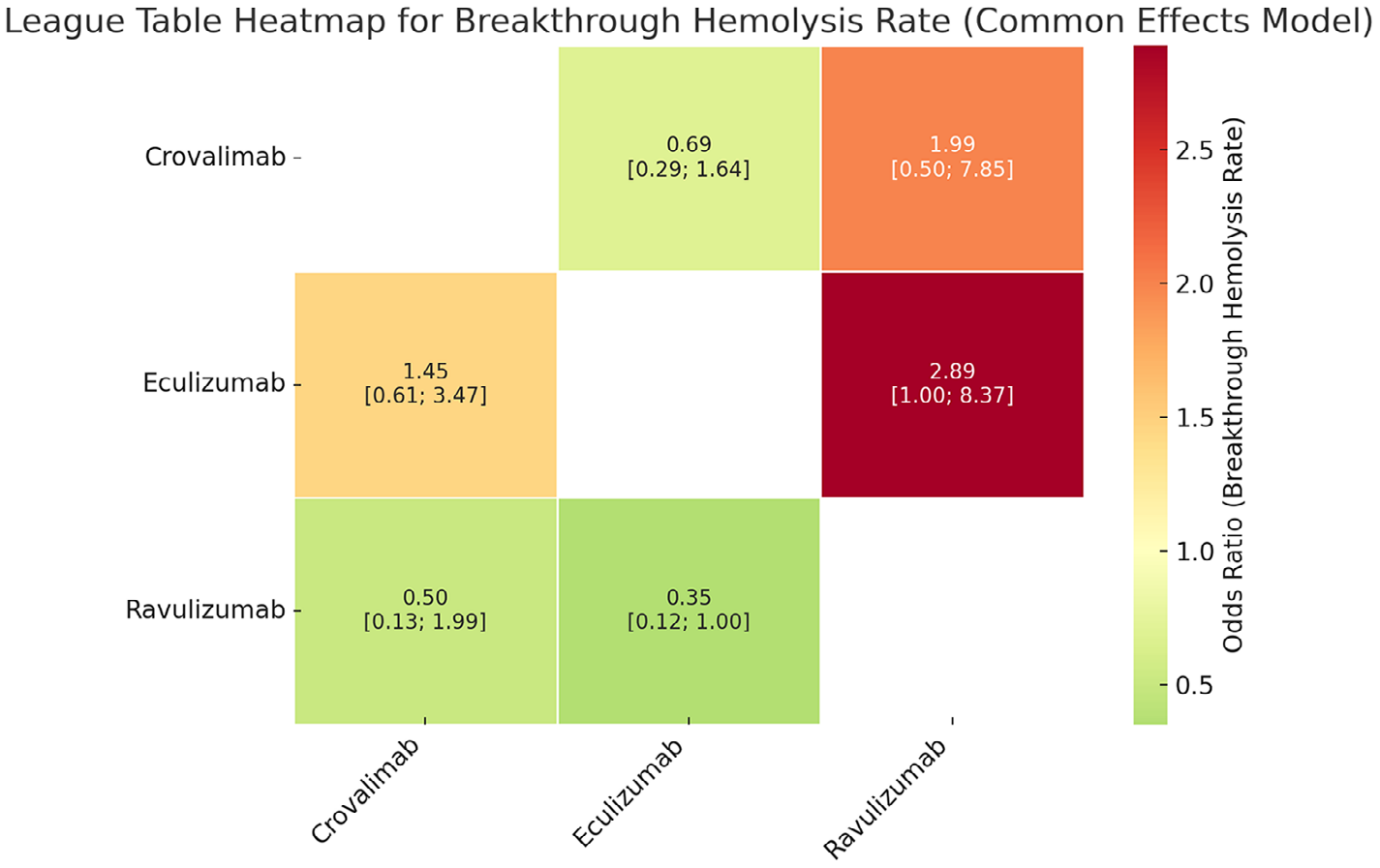
Table for breakthrough haemolysis rate.

- *Total number of red cells transfusions*: In the NMA of two randomized trials evaluating for the total red blood cell transfusions, Both ravulizumab and eculizumab were associated with fewer RBC transfusions as compared to placebo, with mean differences of –8.80 (95% CI: –10.22 to –7.38) and –8.00 (95% CI: –8.32 to –7.68), respectively. However, there was no statistically significant difference between ravulizumab and eculizumab (MD = –0.80, 95% CI: –2.18 to 0.58) (Figure 7).
- *Breakthrough haemolysis*: Breakthrough haemolysis rates were analysed across two RCTs. The findings indicated no statistically significant difference in breakthrough haemolysis rates among the agents (Figure 8).

**FIGURE 9:**
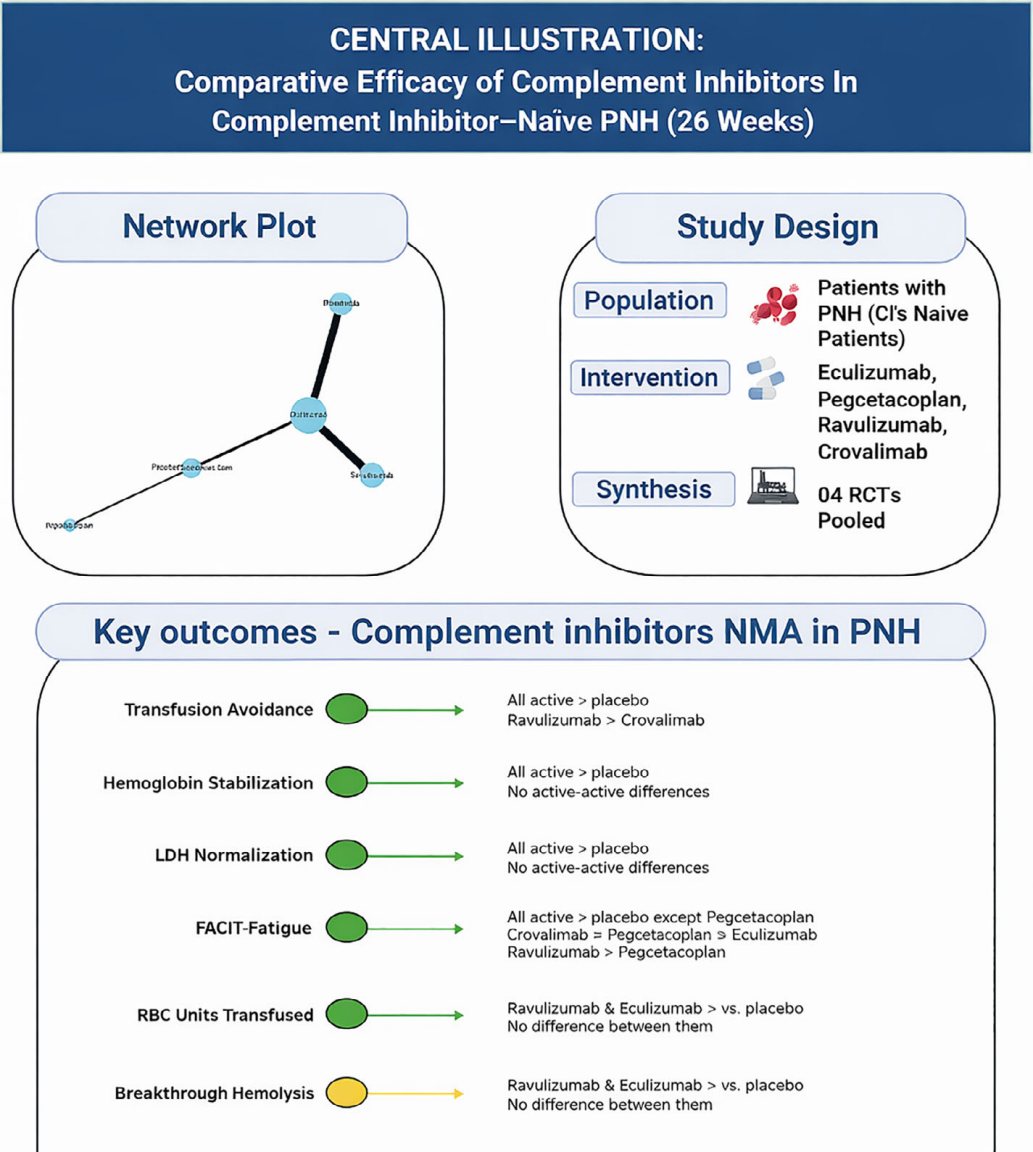
Central illustration summarizing the concluded effects from the network meta‐analysis comparing active complement inhibitors across all reported outcomes.

All NMA forest plots for primary and secondary outcomes are provided in the Supporting Information S2. Figure 9 represents a central illustration summarizing the concluded effects from the NMA comparing active complement inhibitors across all reported outcomes.

## 4 Discussions

As far as we are aware, this systematic review with an exploratory NMA is the first that summarizes randomized evidence comparing available complement inhibitors in PNH patients who have not yet received complement inhibitor treatment. Comparing placebo or supportive care, all evaluated agents demonstrated benefit across key haematologic and patient‐reported outcomes at 26 weeks. However, due to the sparse nature of the evidence network and reliance on indirect comparisons, the findings should be interpreted as hypothesis‐generating rather than definitive comparative evidence.

From our findings, it is clearly visible that point estimates suggested differences in certain outcomes comparing to placebo, while most comparisons between active treatments were not statistically significant, and confidence intervals were wide [13, 14, 15]. Notably, transfusion avoidance appeared numerically higher with some agents; however, this outcome is strongly influenced by baseline transfusion burden, which differed substantially across trials and could not be adjusted for. As a result, apparent differences in transfusion avoidance between active treatments should not be interpreted as evidence of true comparative superiority. Overall, our findings provide an exploratory synthesis of available randomized evidence, highlighting similarities in treatment effects across complement inhibitors and areas where comparative data remain limited in the absence of direct head‐to‐head trials [19].

Similarly, for patient‐reported fatigue, improvements with crovalimab, ravulizumab and eculizumab exceeded the established minimum clinically important difference for FACIT‐Fatigue, indicating clinically meaningful benefit versus placebo. Although statistically significant differences were observed in some indirect comparisons between active agents, the magnitude of these differences was modest and based on indirect evidence from a sparse network. Therefore, their clinical relevance remains uncertain.

In the previous review and meta‐analyses articles, they have focused largely on comparisons of individual agents versus placebo, without comparing the efficacy of the different complement inhibitors with each other or bringing in the newly approved proximal complement inhibitors such as pegcetacoplan or crovalimab. A meta‐analysis of six interventional studies, for instance, by Zhou et al., already showed that eculizumab has an advantage over placebo in significantly reducing haemolysis, LDH levels, improving haemoglobin and lowering the need for transfusions in these patients [20]. Similarly, there is another review article by Lee et al., which pooled data from both double‐arm trials and single‐arm trials in treatment‐naive patients, demonstrating improvement in LDH levels and mean haemoglobin levels within 26 weeks [5]. Therefore, the prior studies have confirmed the superiority of eculizumab over placebo, but they lacked head‐to‐head comparison with the other agents. A recent systematic review by Risitano et al. acknowledged this unmet need but did not perform an exploratory NMA [19]. We included all four complement inhibitors currently approved for PNH in our analyses, highlighting both potential similarities in treatment effects and the limitations of the available comparative data, while acknowledging that additional therapies are approved in certain jurisdictions but were not eligible for inclusion. Overall, the findings underscore that while complement inhibition is effective in treatment‐naïve PNH, current randomized evidence is insufficient to support firm conclusions regarding relative efficacy among active agents. Adequately powered head‐to‐head randomized trials and longer‐term comparative studies are needed to better define differences in efficacy, safety and patient‐reported outcomes.

### 4.1 Strengths of the Analysis

There are many evident strengths to this NMA. It includes only RCTs to ensure high internal validity and minimize bias. By only including double‐armed RCTs in its chosen studies and excluding observational or single‐arm studies, the analysis also upholds methodological rigor and supports causal inference. Inclusion was restricted to complement inhibitor naive patients, enhancing comparability across studies. Plus, the direct comparability between studies, which was made possible by the evaluation of all trial outcomes at a single, consistent timepoint of 26 weeks, was another important aspect of the analysis that contributed to its strength. Efficacy (transfusion avoidance, haemoglobin stabilization, LDH normalization), patient‐centred measures (FACIT‐Fatigue), safety (serious adverse events) and healthcare utilization (total RBC transfusions) were among the many clinically significant outcomes that were examined in order to give the results as much practical relevance as possible. Importantly, the exploratory network meta‐analytic framework allows indirect comparison of therapies in the absence of head‐to‐head trials, while transparently acknowledging the limitations imposed by a sparse evidence network. The reliability of the results was further reinforced by the use of a strong frequentist statistical framework that combined the graph‐theoretical approach with common‐effects modelling. PRISMA guidelines, detailed reporting of search strategies, risk of bias assessments and the use of visual summaries such as PRISMA diagrams, league tables and network plots all contributed to the study's transparency and reproducibility.

### 4.2 Limitations

Due to the sparse network formed by the small number of included RCTs (*n* = 4), effect estimates' accuracy and statistical power are reduced. The small number of trials also prevented official evaluations of inconsistency and limited our ability to conduct sensitivity or subgroup analyses to examine heterogeneity, even though the network was linked.

Subtle variations in the definition and measurement of certain secondary outcomes (e.g., breakthrough haemolysis) across trials may impact the comparability of effect sizes. Importantly, some outcomes were subject to substantial cross‐trial confounding. Furthermore, some comparisons of treatments only used indirect data, which may not be as reliable as comparisons between heads. Furthermore, due to the small number of studies, rankings based on *P*‐scores should be interpreted cautiously and should be regarded as descriptive rather than conclusive.

Generalizability may also be affected as the included trials differed slightly in terms of transfusion practices, regional populations and baseline patient characteristics. Finally, the small number of included studies made it impossible to assess small‐study effects and publication bias. Thus, even though this NMA offers a framework for exploratory comparison, larger networks and more high‐quality RCTs are required to validate these results.

## 5 Conclusion

In the absence of head‐to‐head trials, this systematic review with exploratory NMA synthesizes available randomized evidence on complement inhibitors in treatment‐naive PNH. While all evaluated agents demonstrated clinically meaningful benefit compared with placebo or supportive care, the sparse evidence network and reliance on indirect comparisons preclude firm conclusions regarding relative efficacy among active treatments. Consequently, observed differences should be regarded as hypothesis‐generating rather than definitive.

## Author Contributions

RI chose the study topic and designed the research framework. S.R provided mentorship and guidance throughout the process. H.M, F.T.M, H.S did study screening and data extraction. T.R conducted the statistical analysis. F.T and A.S.T contributed to manuscript drafting and editing. All authors reviewed and approved the final manuscript.

## Funding

The authors have nothing to report.

## Conflicts of Interest

The authors declare no conflicts of interest.

## Supporting information




Supporting File 1

## Data Availability

The data that support the findings of this study are available from the corresponding author upon reasonable request.
