# Supplementary figures and images for: Comparative Efficacy of Complement Inhibitors in Complement Inhibitor–Naïve PNH: A Systematic Review With Supportive Exploratory Network Meta‐Analysis of Randomized Trials
Source: EJHaem. 2026 Feb 24;7(1):e70250. doi: 10.1002/jha2.70250 (PMC12931153; doi:10.1002/jha2.70250)

**SUPPLEMENTARY MATERIALS:**

**S1: ROB Assessment**

**
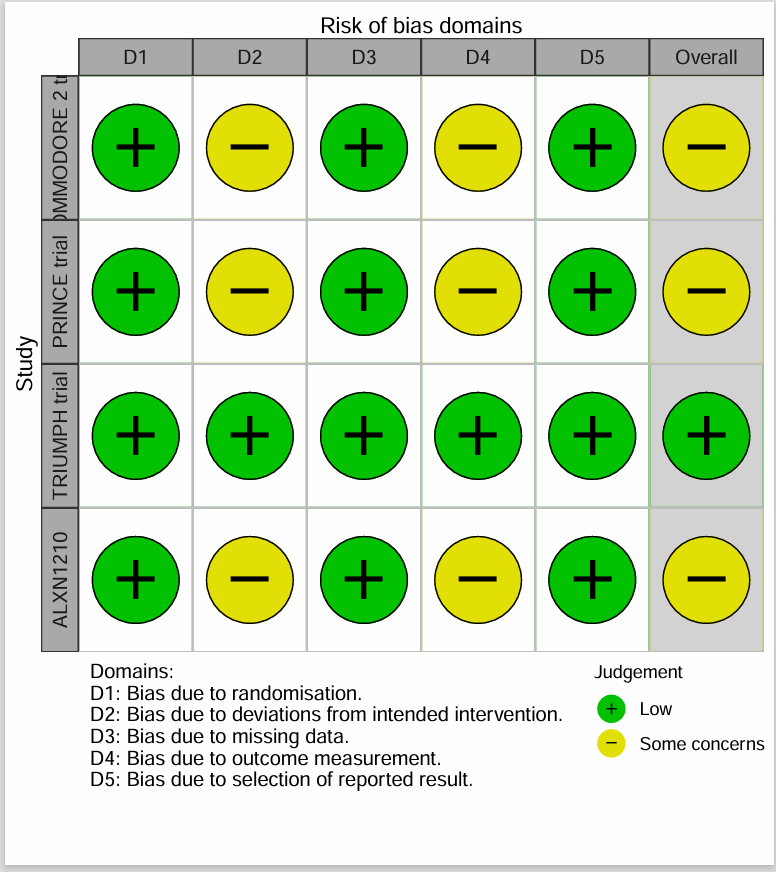
**

**S2: Forest plots**

**
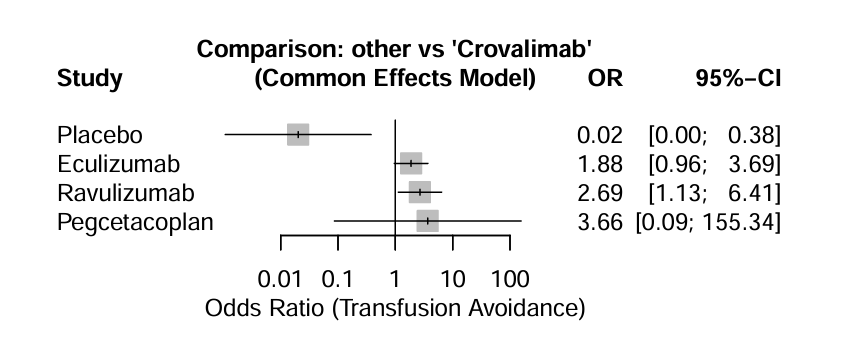
**

**
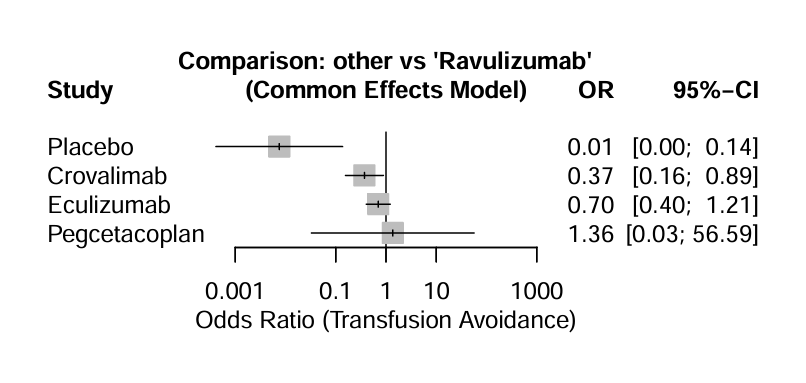
**

**
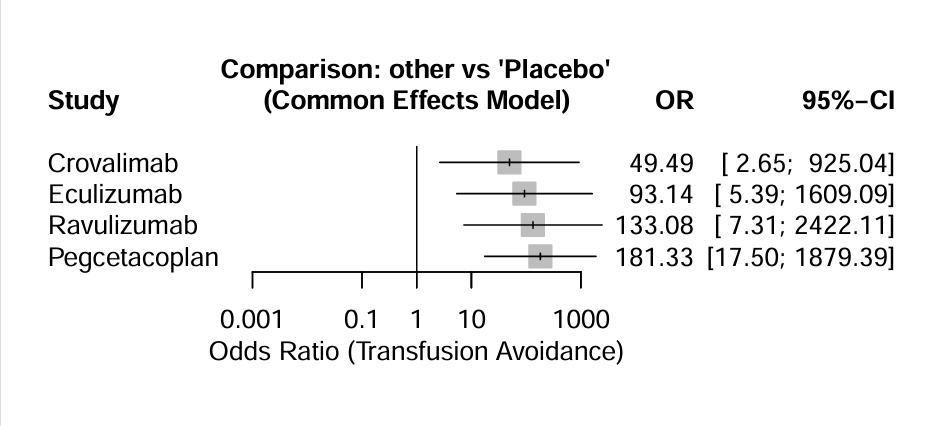
**

**
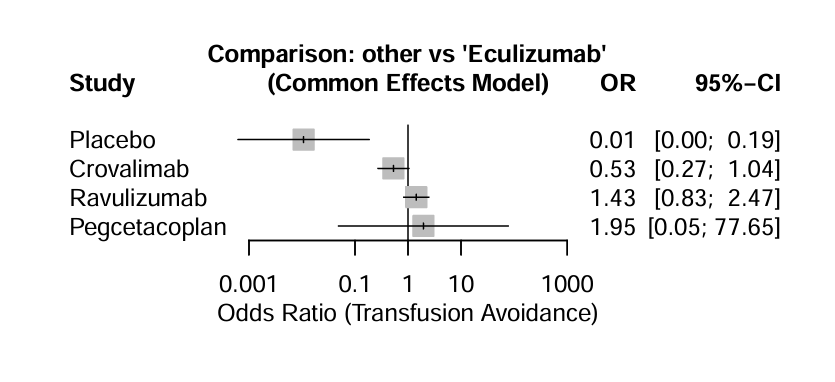
**

**
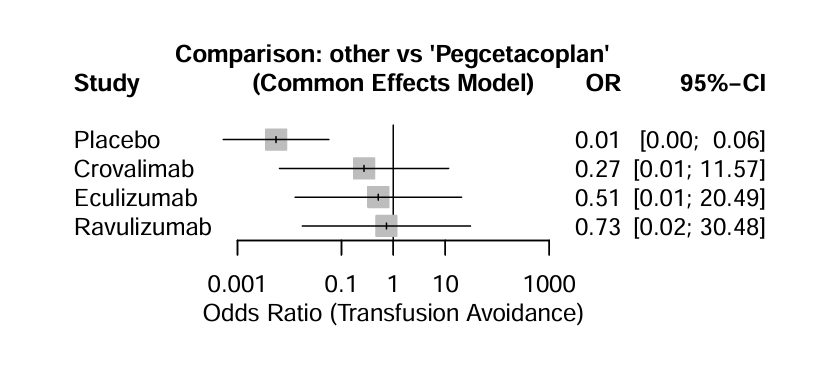
**

**
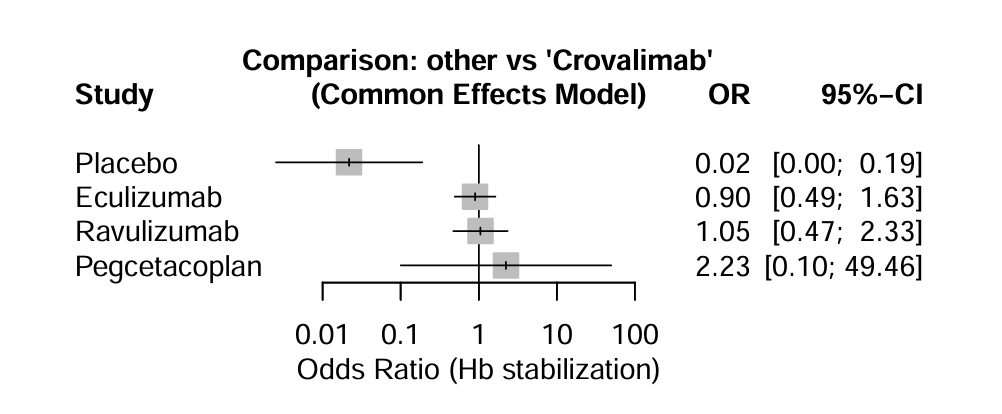
**

**
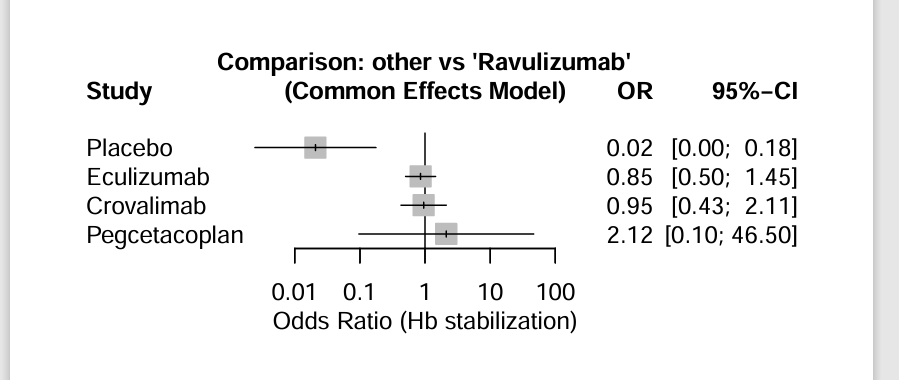
**

**
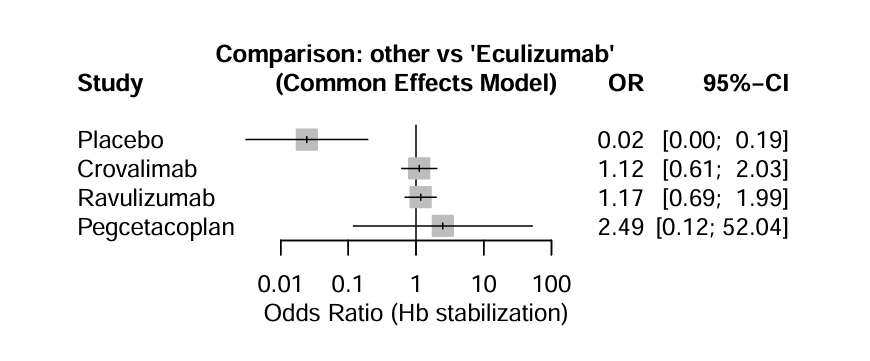
**

**
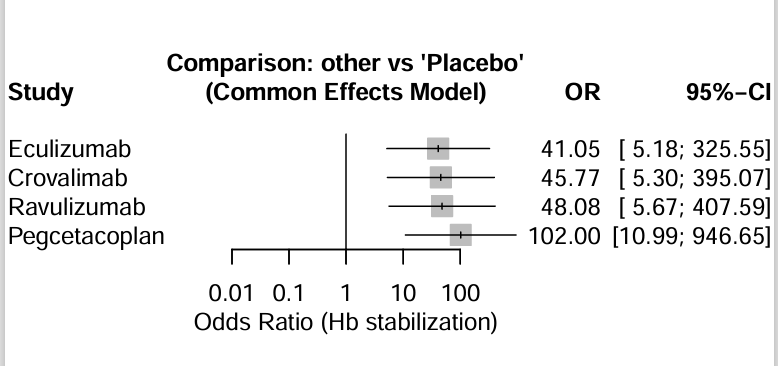
**

**
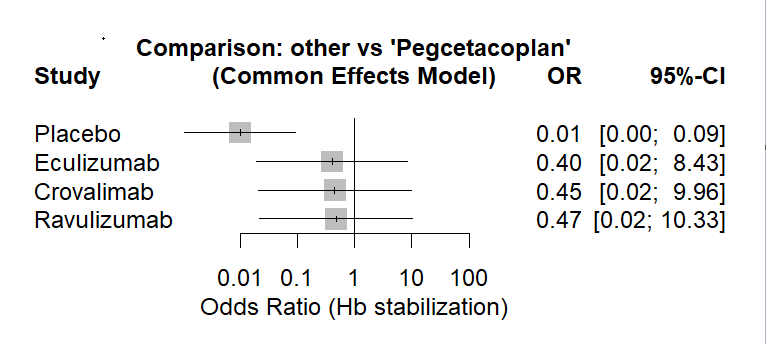
**

**
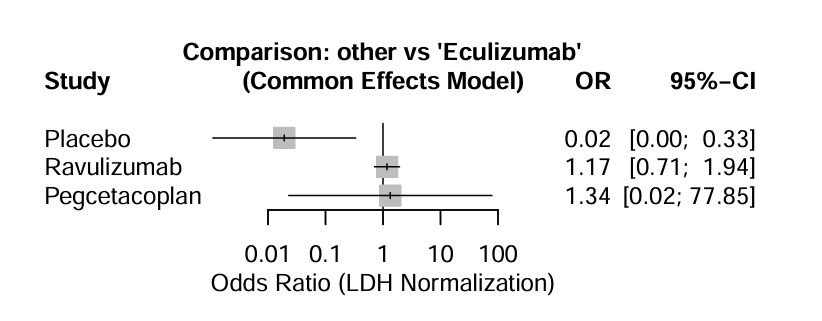
**

**
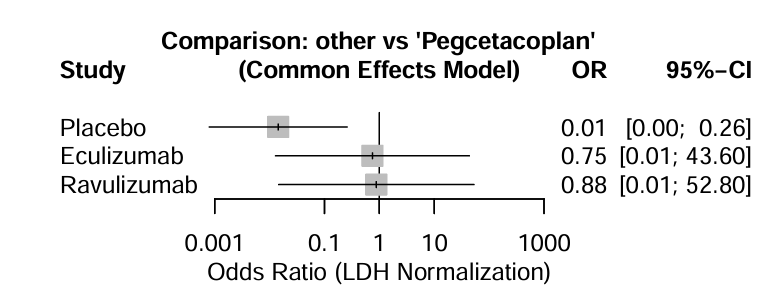
**

**
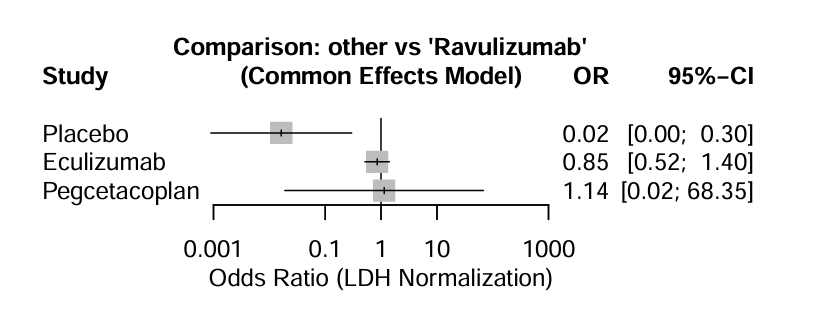
**

**
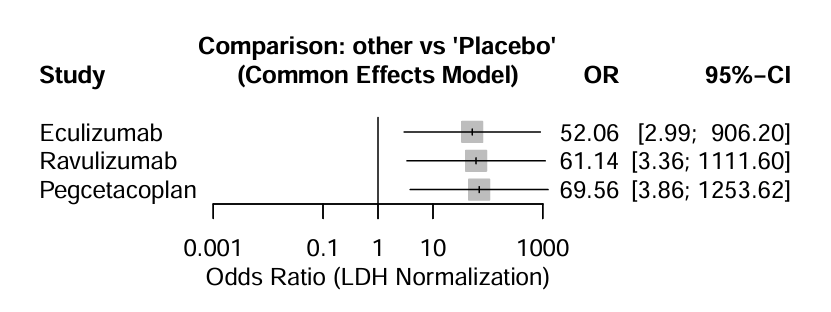
**

**
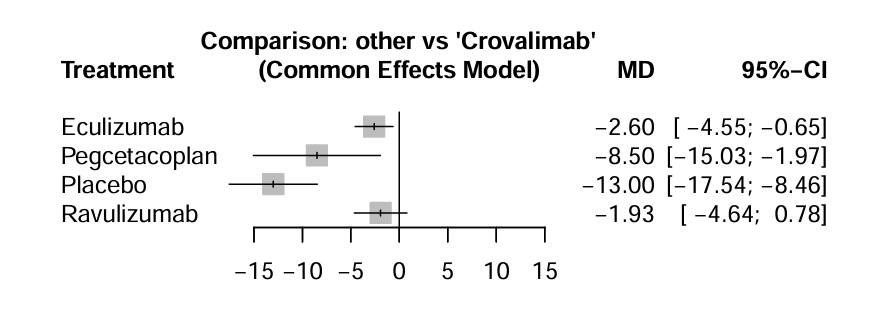
**

**
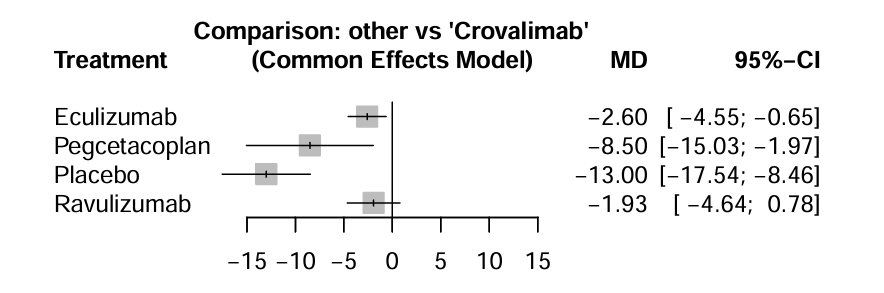
**

**
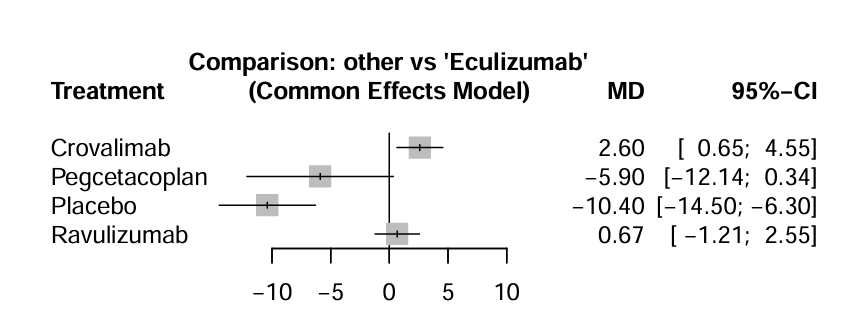
**

**
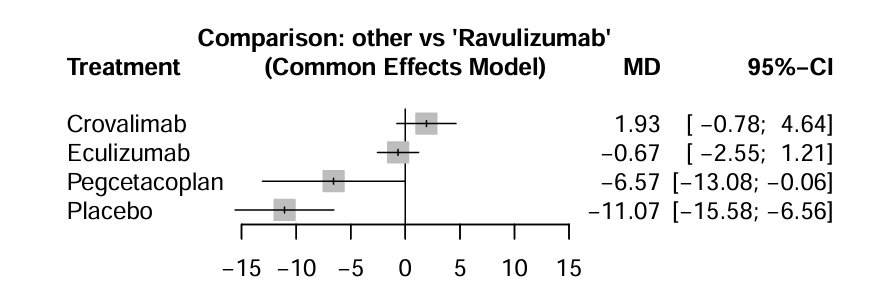
**

**
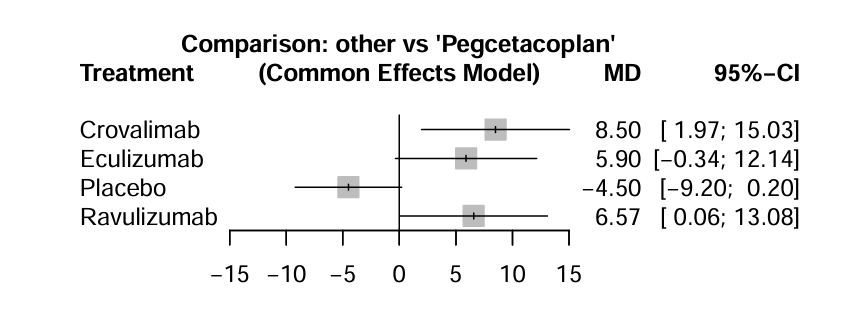
**

**
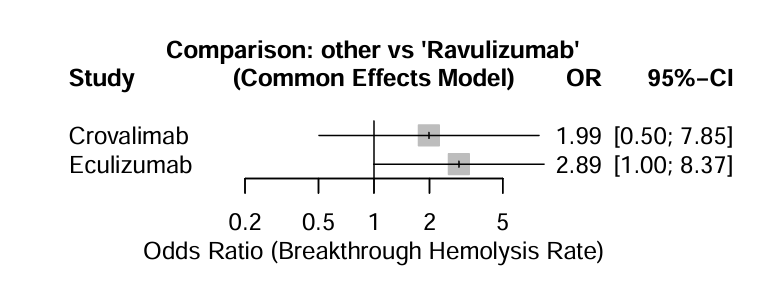
**

**
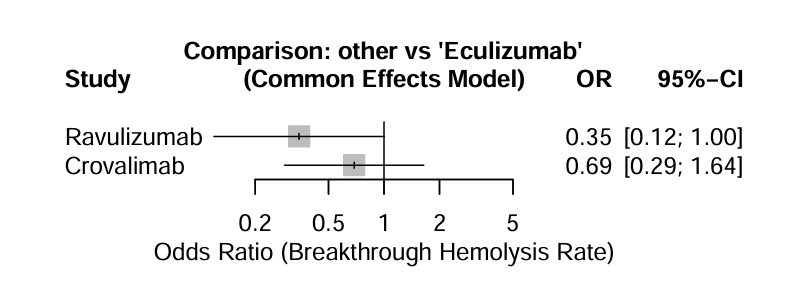
**

**
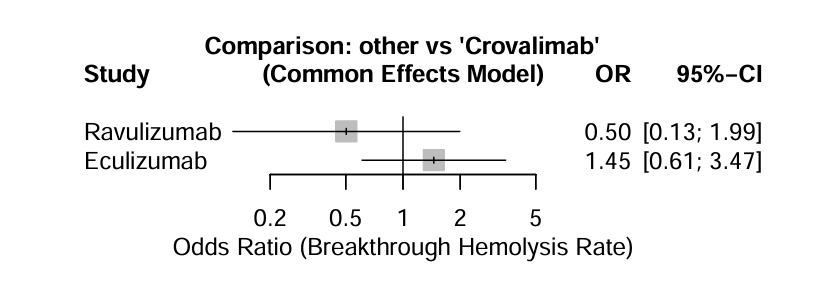
**

**
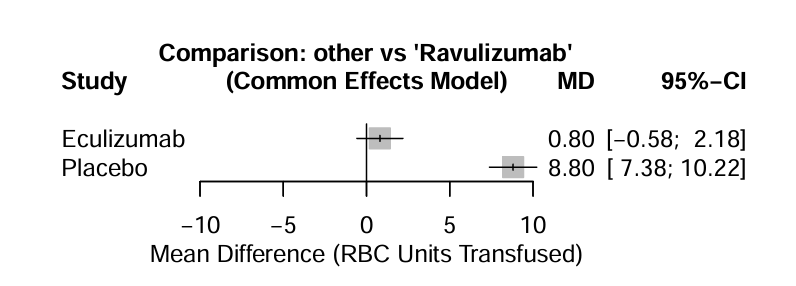
**

**
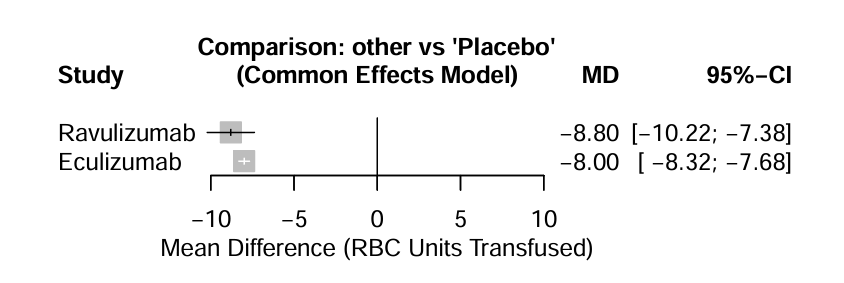
**

**
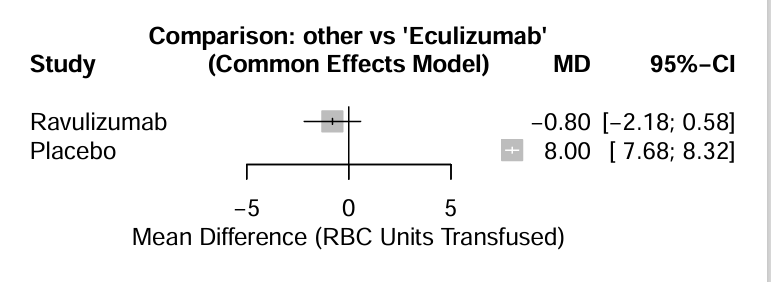
**

Supplement: Supplementary file 1 — Supporting File 1 [file JHA2-7-e70250-s001.docx]
